# Supplementary figures and images for: An Efficient and Frequency-Scalable Algorithm for the Evaluation of Relative Permittivity Based on a Reference Data Set and a Microstrip Ring Resonator
Source: Sensors (Basel). 2022 Jul 26;22(15):5591. doi: 10.3390/s22155591 (PMC9370874; doi:10.3390/s22155591)

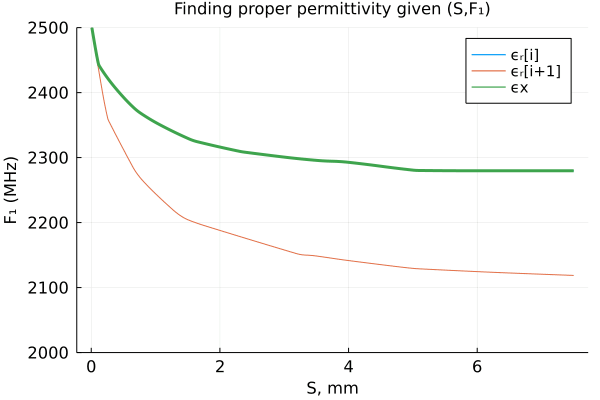

Supplement: Supplementary file 1 [file sensors-22-05591-s001.zip › sensors-1794074-supplementary.gif]
